# Leak or link? the overrepresentation of women in non-tenure-track academic positions in STEM

**DOI:** 10.1371/journal.pone.0267561

**Published:** 2022-06-08

**Authors:** Stephanie Rennane, Hannah Acheson-Field, Kathryn A. Edwards, Grace Gahlon, Melanie A. Zaber

**Affiliations:** 1 Economics, Sociology, and Statistics, RAND Corporation, Arlington, Virginia, United States of America; 2 Pardee RAND Graduate School, RAND Corporation, Santa Monica, California, United States of America; 3 Johns Hopkins University School of Public Health; Baltimore, Maryland, United States of America; 4 Economics, Sociology, and Statistics, RAND Corporation, Pittsburgh, Pennsylvania, United States of America; Iowa State University, UNITED STATES

## Abstract

This paper examines gender variation in departures from the tenure-track science, technology, engineering, and math (STEM) academic career pathway to non-tenure-track academic careers. We integrate multiple data sources including the Survey of Earned Doctorates and the Survey of Doctorate Recipients to examine longitudinal career outcomes of STEM doctorate women. We consider three types of careers after receipt of a PhD: academic, academic non-tenure-track, and non-academic positions. We find that STEM women are more likely to hold academic non-tenure-track positions, which are associated with lower job satisfaction and lower salaries among men and women. Explanations including differences in field of study, preparation in graduate school, and family structure only explain 35 percent of the gender gap in non-tenure-track academic positions.

## Introduction

For many individuals pursuing graduate degrees in the science, technology, engineering, and math (STEM) fields, the ideal culmination of these academic pursuits is a tenured faculty position. To reach this goal, scholars must go through many stages in the career pathway after achieving the PhD: obtaining a post doctorate position in many fields, followed by a tenure-track position. However, women in STEM are less likely to finish PhDs, less likely to get tenure-track positions if they do finish, and less likely to get tenure if they do get an initial position [1–5]. The gender imbalance in tenured STEM faculty has significant consequences for the future of STEM. Women and other underrepresented groups have been shown to perform better in settings with colleagues, role models, and peers from similar backgrounds [6, 7]. As a result, a less diverse STEM academic workforce hinders the progress and success of future female and minority scholars. Underrepresentation of women and minorities also leads to a less diverse STEM workforce producing research output [8–10]: a less inclusive academy could result in less innovation and less scientific debate.

At the same time, the traditional STEM career pathway is declining in relevance, as many individuals in STEM pursue alternative career paths [11]. Furthermore, the existing explanations for the underrepresentation of women have focused on factors that could “push” women out of the academic career path [12, 13], such as sexism or caring for children, rather than an assessment of job characteristics and scholars’ preferences over them. Finally, with the notable exception of a recent paper by Ding and coauthors on gender-based pay disparities in academia [14], the existing literature has overlooked an important subgroup of STEM researchers which warrants closer attention: PhD researchers who hold non-tenure-track positions within academia. In this paper, we examine this latter group in closer detail, and focus on measuring differences in the characteristics and details of job attributes and preferences over these attributes, rather than on other external factors which may influence men and women’s decisions to pursue certain careers.

We take a closer look at the patterns and consequences of gender differences in pathways in STEM careers, starting from the point of receipt of the PhD. In particular, we explore transitions in and out of tenure-track and non-tenure-track academia for men and women in STEM. We find that women in STEM are more likely to stay in academic positions than men, but they are disproportionately represented in non-tenure-track academic jobs. While this disproportionate representation of women in non-tenure-track positions may not be visible when perusing faculty directories or in published department demographic statistics (which may combine tenure-track and non-tenure-track faculty), this distinction has significant implications for science, a scholar’s career and quality of life. We explore the implications of a higher share women starting, transitioning and remaining in non-tenure-track academic positions from both an individual and societal perspective. On one hand, women in these positions may teach and conduct research similarly to tenure-track faculty, so the distinction may not make a large difference in terms of women’s broader contributions to STEM fields. On the other hand, if women are constrained in their job search and non-tenure-track positions reflect a suboptimal outcome, this disproportionate sorting would have consequences both for the overall output, productivity and diversity of STEM fields and for women’s careers.

We examine longitudinal outcomes of women in STEM and explore how career pathways vary by gender with an analytic data file constructed from several data sources. We integrate data from the Survey of Earned Doctorates (SED) [15], the Survey of Doctorate Recipients (SDR) [16], the Integrated Postsecondary Education Data System (IPEDS) [17] and the National Research Council (NRC) [18]. The SED is an annual census conducted of all graduates earning a research doctorate in a given year. A subset of individuals from the SED are followed longitudinally in the SDR, allowing us to observe doctorates at various phases in their careers. The data from IPEDS and NRC provide data on institutional characteristics, which we link to the graduating institution listed in SED to provide more detail about the environment where individuals earned their doctorates. We focus on individuals who received their PhD during or after 2000 and track individuals through 2015, resulting in a sample of 51,236 individuals (See the supplement for details).

We consider three types of careers after receipt of a PhD: the academic, academic non-tenure-track (e.g., staff scientists or lecturers), and non-academic pathways (e.g., jobs in industry or in government). STEM fields are defined based on the National Science Foundation’s (NSF’s) Science, Engineering and Health (SEH) definition used in the SED and SDR. This a slightly broader definition of STEM which includes the physical, life, social, engineering, and mathematical sciences as well as health science (e.g., physiology and nursing science), as well as some education and business and management degrees. We identify gender based on self-reports in the survey data. The resulting data set contains rich information about STEM graduates over fifteen years, allowing us to observe various career pathways and to explore job and personal characteristics and preferences associated with each of them. Our aims are (1) to better understand the nuances of gender-based differences in deviations from the STEM tenure track, including movement into non-tenure-track academic careers, (2) to explore the consequences of shifts into non-tenure-track positions, and (3) to explore potential factors that may contribute to gender-based differences in career trajectories within STEM.

## Materials and methods

### Materials

Our analyses combine several data sources. First, we use the Survey of Earned Doctorates (SED), which is an annual census conducted of all graduates earning a research doctorate in that year. The SED solicits information on doctorate field, demographics, forms of support during graduate school, and employment intentions. Second, we use the Survey of Doctorate Recipients (SDR), a biennial longitudinal survey following individuals with a doctorate in a STEM field over time. The SDR asks respondents about changes in employment, field, work activity, job amenities (e.g., salary), satisfaction, preferences over job activities, and family information since the prior wave. Each wave of the SDR adds a new subset sample of individuals in the SED who earned a doctorate in a STEM field in the last two years. We utilize the restricted-use SED-SDR data under a license from the NSF National Center for Science and Engineering Statistics. These data are anonymized to protect survey respondent confidentiality and respondents consented to the use of their data in research when filling out the survey.

We additionally link in characteristics on individuals’ graduate PhD-granting institutions. First of all, we link information from the Integrated Postsecondary Education Data System (IPEDS) and the National Research Council (NRC). IPEDS contains key information about postsecondary institutions including whether its ownership (e.g., public, private non-profit, or for-profit), university revenue, and admissions selectivity. We also incorporate IPEDS data on the diversity of the institution, including information about the demographics (e.g., race and gender) of staff, tenure-track faculty, new hires, enrollees and completions. Unfortunately, this information is only available at the institution level, not separately by degree program or department. We match data from IPEDS to the year in which the individual received their doctorate.

We incorporate additional information about the institution from the National Research Council. This data includes detailed information about research and student activity including the percent of faculty with grants or other awards, publications and citations, share of first year graduate students with full support, diversity (share of graduate students who are female and minority) and median completion times for PhDs. This information is available at the program level, which is in fact more detailed than what we are able to observe in the SDR. As a result, we take the average of these statistics within a given field for each institution, and match to the SDR at the field*institution level. While this data is available in finer categories within an institution, it only captures these characteristics in 2006. As a result, we take the 2006 averages and match them to the SDR by institution and field.

The linked SED-SDR sample captures some graduates from nearly all STEM-related fields. We define STEM as fields included in the SDR, which is based on the NSF definition of SEH and includes the physical, life, social, engineering, and mathematical sciences as well as health science (e.g., physiology and nursing science), as well as some education and business and management degrees. See https://www.nsf.gov/nsb/sei/infographic2/#fields-of-degree for more details on the degrees included in the SEH definition.

Importantly, however, because the SDR selects only STEM graduates, the graduates in our sample from fields such as humanities and education are subset to the subfields within these disciplines categorized as STEM by the SDR (e.g., high school education with a specialization in biology). While the SED and SDR both contain information about graduates with degrees as early as the 1950s, we limit our sample to respondents who graduated with their first doctorate in 2000 or later, tracking respondents through the 2015 wave of the SDR. This restriction ensures a contemporary sample of doctorates making decisions about employment in the current academic and employment environment. In total, we have a sample of approximately 52,000 STEM doctoral recipients, whom we can follow for varying durations depending on the year of degree and completeness of subsequent waves in the SDR.

The resulting data set contains incredibly rich, longitudinal information about STEM graduates over for about fifteen years, allowing us to observe entry and exit from academia, and to explore job and personal characteristics and preferences associated with these decisions. However, there are some limitations. First, the NSF expanded the SDR sample for the 2015 wave (the most recently-available wave of the restricted-use data), and 59 percent of all respondents in our sample are first surveyed in 2015, changing the composition of the sample. Secondly, detailed measures of job satisfaction (e.g., satisfaction with particular job components, like independence or salary), were not included in the 2006 and 2008 surveys.

Our analyses focus on three main career paths after receipt of a PhD: academic tenure track, academic non-tenure-track, and non-academic positions. We identify positions in each group based on survey responses in the SDR to a question asking respondents to describe their employer type, and their tenure status at their academic institution (if they indicated that they were employed by an academic institution). The responses to this question are “Not applicable: no tenure system at this institution,” “Not applicable: no tenure system for my position,” “Tenured,” “On tenure track but not tenured,” and “Not on tenure track.” We identify post doctorate positions in the SDR based on whether a respondent indicated that they were employed by college or university, medical school, or university-affiliated research institute and that they held a post doctoral position at this university. We identify post doctorate positions in the SED based on whether a graduate indicated that they intended to take a post doctoral fellowship or post doctoral research associateship position. Individuals in post doctorate positions are not counted in either the academic non-tenure-track or in the tenure track categories. We identify the primary activities being conducted by individuals in non-tenure-track positions using reported information about respondents’ primary work activities. Individuals are classified as being in a non-tenure-track position focused on research if they report research as a primary activity, but not teaching (and vice-versa for teaching). We create a group of individuals who report both research and teaching as primary activities as well as neither research nor teaching.

### Methods

We conduct descriptive analyses using STATA version 15 comparing men and women at various points in time after completion of the PhD and across job type. With the exception of the hazard analysis, all models leverage variation between PhD-holders, rather than within an individual. We first examine trends in the characteristics of men and women at the time of PhD completion to understand what the population looks like at the beginning of the pipeline. Then, we compare job characteristics and activities, job satisfaction and job outcomes among men and women in different job types both at the time we first observe them in the SDR and at a fixed point later in their careers, 7–9 years after PhD completion. We implement a series of linear regression models to understand how job satisfaction varies between men and women in different types of career paths (academic, academic non-tenure-track, and non-academic). To do this, we estimate a regression model as follows:

$$Y_{it}=\alpha +\sum_{n=1}^{N}\beta_{n}I_{int}+\varphi_{f}+\delta d_{it}+\varepsilon_{it}$$ (1)

Where *Y_it_* is a measure of job satisfaction (either overall, or satisfaction with a specific job component, such as advancement, location or salary) for individual i at time t; $I_{int}$ are a series of indicators for individuals in a specific job type-gender category n, *φ_f_* are fixed effects for field of study, and *d_it_* is a measure of duration since the time of graduation with the PhD. In our primary specification, we focus on examining satisfaction 7–9 years after graduation. The omitted category in the series of indicators is tenured men, so the coefficients on the indicators *β_n_* reflect how job satisfaction varies for each group relative to tenured men.

After exploring differences in job activities and satisfaction, we explore factors that could explain the observed sorting of women into non-tenure-track academic positions. To do this we implement another linear regression model as follows:

$$Y_{it}=\alpha +\beta F_{i}+\varphi_{f}+I_{i}\theta +X_{it}\lambda +\varepsilon_{it}$$ (2)

In Eq (2), *Y_it_* is an indicator for being in a non-tenure-track position; *F_i_* is an indicator for females, and *I_i_* and *X_it_* are a series of institution and job characteristics, respectively, and *φ_f_* again are fixed effects. The coefficient *β* indicates whether women have a significantly higher or lower likelihood of being in non-tenure-track positions in STEM, conditional on the other factors included in the regression. As shown in Table 3, we first estimate the regression including only the *F_i_* indicator, and then subsequently add each additional category of factors (e.g., field, then field and institution factors, then field, institution and family factors) to examine the extent to which these other factors may explain any of the observed difference in the share of women in non-tenure-track positions as reflected in the *β* coefficient.

## Results

We begin by examining trends in the characteristics of men and women at the time of PhD completion to understand what the population of STEM doctorates looks like upon completing their education and finding their first position. Table 1 presents characteristics for men and women overall at the time of PhD completion. Approximately 36 percent of women report that their first job will be in a U.S. university or medical school, compared to 31 percent of men. On the other hand, 20 percent of men report a first job in industry compared to 10 percent of women. Approximately 28 percent of women and 24 percent of men do not report a first job at the time of PhD completion, likely due to discrepancies in the timing of the job market in various fields relative to when the survey was completed. A higher share of STEM women than men earned degrees in biological or life sciences (29 vs 23 percent), psychology (20 vs 6 percent) and social sciences (14 vs 11 percent), while a higher share of STEM men earned degrees in engineering (28 vs. 10 percent) and physical science (18 vs. 11 percent).

**Table 1:** Comparison of characteristics at the time of PhD between all men and women.

	Women	Men	P-value
**Post-PhD Job (from SED)**			
U.S. University/Medical School	0.36	0.31	<0.001
U.S. Government	0.07	0.07	0.363
Industry/Business	0.10	0.20	<0.001
Not for Profit	0.06	0.05	0.003
**Field**			
Bio/life science	0.29	0.23	<0.001
Computer/info science	0.02	0.05	<0.001
Engineering	0.10	0.28	<0.001
Health	0.10	0.03	<0.001
Math/Stat	0.03	0.05	<0.001
Physical science	0.11	0.18	<0.001
Psychology	0.20	0.06	<0.001
Social Science	0.14	0.11	<0.001
**PhD Institution Characteristics**			
Average pubs per allocated faculty	1.66	2.02	<0.001
Number of programs within fields	1.48	1.29	<0.001
% of faculty with grants	0.71	0.77	<0.001
% of first year students with full support	0.87	0.88	<0.001
% completed PhD within 6 years	0.46	0.47	0.001
Percent of institution with academic plans	0.57	0.51	<0.001
Median time to degree	5.70	5.49	<0.001
**Personal preparation**			
Years in grad school	8.34	7.98	<0.001
Any debt	0.51	0.44	<0.001
Had a postdoc	0.30	0.31	0.253
Research assistant experience during grad school	0.54	0.61	<0.001
Grant funded during grad school	0.29	0.23	<0.001
Fellowship funded during grad school	0.53	0.47	<0.001
**Demographics at time of PhD**			
Married	0.47	0.52	<0.001
Age	34.26	33.46	<0.001
Children living at home	0.32	0.50	<0.001
**At first observation in Survey of Doctoral Recipients**			
Academic—TT	0.21	0.21	0.156
Academic not TT	0.20	0.13	<0.001
Academic—not TT, teaching	0.06	0.04	<0.001
Academic—not TT, research	0.05	0.05	0.022
Academic—not TT, both	0.02	0.02	0.506
Academic—not TT, neither	0.07	0.03	<0.001
Postdoc	0.12	0.12	0.477
Nonacademic	0.47	0.54	<0.001
Salary	73,855	86,734	<0.001
Job in STEM field	0.70	0.84	<0.001
Observations (unwt)	24232	27004	

Notes: Average demographic, field, institution and career characteristics at the time individuals complete their PhDs, separately for men and women. Analyses based on data from the National Survey of Earned Doctorates and National Survey of Doctoral Recipients. Means calculated using survey weights. *p* values reported from a two-tailed *t*-test of whether the difference in means between men and women is statistically significant.

We next consider characteristics of the institutions where men and women earned their degrees, and factors related to their preparation before and during graduate school. Institutions for men and women have similar shares of graduate students with full first-year support and similar completion rates within six years. Women attend schools with fewer average publications per faculty (1.6 vs. 2) and longer median time to degree (5.7 vs. 5.5 years), which could either indicate differences in the relative quality of the institutions attended by women and men, or simply reflect differences in the composition of fields pursued by men and women. On average, women attend universities where a higher share of students intend to pursue academic careers (57 vs. 51 percent), and with a higher number of programs offered within fields (1.5 vs. 1.3). These characteristics are all noisy measures of institution quality and the magnitude of the differences is small. However, the trends do not suggest that women systematically attend universities which afford them fewer opportunities in terms of available courses of study, faculty quality or student support.

There are more marked differences in terms of pre-degree experiences. On average, women spend about half a year longer in graduate school, are more likely to be funded by grants or fellowships during graduate school and, perhaps consequently, are less likely to have research assistant experience during graduate school (54 vs. 61 percent). They are also more likely to have debt at the time of PhD completion (51 vs. 44 percent). In terms of family characteristics, women are slightly less likely to be married (47 vs. 52 percent) and significantly less likely to have children (32 vs. 50 percent) at the time of their PhD completion.

Despite these commonalities and minor differences among STEM PhD-holders, we see substantial, gender-correlated differences in placement outcomes. Table 1 also presents the overall sorting into job type and characteristics at the time of the first observation in the SDR in seven categories: tenure-track academic, non-tenure-track academic (in positions focused on teaching, research, both or neither), non-academic, and postdocs. Equal shares of women and men report being in tenure-track academic jobs and postdocs, while men are more likely to be working in in non-academic positions at the time of first observation in SDR (54 vs. 47 percent). A higher share of women report being in any non-tenure-track academic job (20 vs. 13 percent). In particular, a higher share of women in positions focused on teaching (6 vs. 4 percent) and positions with a focus that is neither teaching nor research (7 vs. 3 percent). Similar shares of women and men are in non-tenure-track positions focused on research or both research and teaching at the first observation in the SDR. The exact number of years since degree at first observation in the SDR varies depending on when individuals are surveyed in the SDR relative to when they completed their degrees, but typically ranges between 2–5 years in our sample.

**Fig 1:**
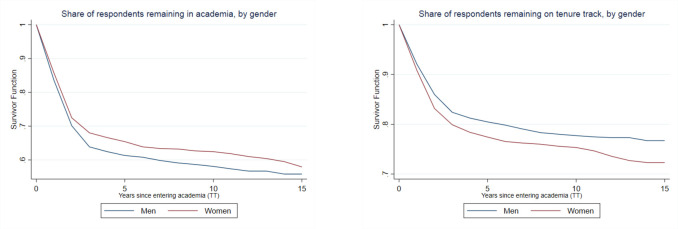
Persistence of men and women remaining in academia and remaining on the tenure track. (A) Share of respondents remaining in academia, by gender. (B) Share of respondents remaining on tenure track, by gender. *Notes*: Survival curves showing the share of men and women who remain in academia overall (A), conditional on not having left academia in prior years; and the share of men and women who remain on the tenure track (B), conditional on not having left the academic tenure track in prior years. Time measured relative to the point when individuals enter the academia. Analyses based on data from the National Survey of Earned Doctorates and National Survey of Doctoral Recipients.

Fig 1A presents a survival curve showing the how likelihood of remaining in academia evolves over the first 15 years after respondents enter academia. The survival curve in Fig 1B shows the likelihood of remaining on the tenure track, for the subset of respondents who we observe remaining in academia. These figures show a pattern consistent with the baseline means in Table 1: women are more likely than men to remain in academia, but conditional on being in academia, they are less likely to start and remain on the tenure track. Furthermore, this pattern is persistent over time, and the gender gap in non-tenure-track positions remains even 15 years after we observe respondents entering academia. Women are more likely to remain in all types of non-tenure-track positions (encompassing those focused on teaching, research, both, or neither) at 3–5 years post-graduation and 7–9 years post-graduation (see S1 Table).

The trends in the gender gap also vary by field. Three to five years after receipt of the PhD, the gender gap in non-tenure-track academic positions is largest in the Biological and Life Sciences (23 percent of women vs. 18 percent of men in non-tenure-track positions) and Health Sciences (25 percent of women vs. 15 percent of men in non-tenure-track positions). There is also a significant gap ranging between 1 and 7 percentage points among Engineering, Physical Science, and Social Science doctorates at 3–5 years after graduation. The gender gaps generally remain consistent when looking 7–9 years after PhD graduation, though there are increases in Biological and Life Sciences and Psychology between 3–5 years and 7–9 years after PhD graduation (S1 Table).

We next explore the implications of women being disproportionately represented in non-tenure-track academic positions throughout their careers. We examine effects on contributions to science (measured by activities performed on the job), as well as effects for women’s careers (measured by overall job satisfaction, satisfaction with specific job components, and salary). Job satisfaction is measured on a Likert scale from 1 to 4 where 4 is “Very satisfied” and 1 is “Very dissatisfied.” We study these outcomes at a fixed point, 7–9 years after PhD completion, to focus on a steady state after the period when most postdocs have been completed.

Job activities vary significantly across job type (Table 2). Individuals in tenure-track academic positions and non-tenure-track positions with an emphasis on research are more likely to report research as a primary activity and are more likely to have work funded by grants. In fact, the highest share of individuals with work supported by grants– 72 percent–are in non-tenure-track research positions. Tenure track academics are most likely to attend professional meetings and participate in professional societies, while non-academics are least likely. Tenured academics and non-tenure-track academic teaching positions supervise the most individuals (approximately 6.5 individuals on average). In general, these patterns suggest activities consistent with the requirements of various job types and are generally similar for both women and men. These statistics do not necessarily shed light on whether individuals in non-tenure-track positions are constrained in their ability to contribute to their fields due to their position, nor whether they took non-tenure-track academic positions due to a lack of other options.

**Table 2:** Job outcomes and activities by job type and gender. SDR stats measured for observations between 7–9 years post-PhD.

	Academic TT—tenured				Academic not, teaching position			Academic not, research position			Academic not, teaching & research		
	Women	Men	P-value		Women	Men	P-value	Women	Men	P-value	Women	Men	P-value
Work funded by grants	0.30	0.39	< 0.001		0.11	0.11	0.875	0.69	0.74	0.127	0.37	0.40	0.682
Primary work activity is research	0.87	0.90	0.014		0.49	0.56	0.077	0.96	0.97	0.670	0.88	0.96	0.007
Number of individuals supervised directly	6.54	6.36	0.744		6.73	6.37	0.745	5.83	3.53	0.186	6.09	5.37	0.419
# of professional meetings attended	0.84	0.87	0.091		0.61	0.63	0.682	0.77	0.72	0.111	0.88	0.87	0.758
# of professional society membership	2.77	2.51	0.002		1.87	1.99	0.390	1.85	1.75	0.294	2.56	2.54	0.884
Working outside field because other job not available	0.29	0.20	0.754		0.13	0.37	0.245	0.41	0.38	0.885	0.92	0.63	0.479
Overall job satisfaction	3.44	3.48		0.106	3.23	3.24	0.848	3.22	3.17	0.341	3.09	3.35	0.003
Observations	925	1114			386	290		445	465		164	159	

*Notes*: Percentage of men and women in each job type who report various job activities, including research, grant funding, supervision of other employees and working outside of the field. Analyses based on data from the National Survey of Earned Doctorates and National Survey of Doctoral Recipients. Means calculated using survey weights. *p* values reported from a t-test of whether the difference in means between men and women is statistically significant.

However, we also examine respondents’ answers to the question of whether they work outside of their fields because no other jobs were available. The share of individuals who report working outside of their field is approximately 40 percent in non-academic positions and does not vary significantly between men and women. However, the share is significantly higher for two types of non-tenure-track positions: those involving both teaching and research and those involving neither teaching nor research. In both of these job types, the share of women who report working outside of their field due to a lack of other available jobs is significantly higher than for men (92 vs. 63 percent in non-tenure-track positions with both teaching and research; 39 vs. 20 percent in non-tenure-track positions with neither teaching nor research). This suggests that at least part of the disproportionate representation of women in non-tenure-track academic positions could result from difficulties finding a suitable position in their field.

**Fig 2:**
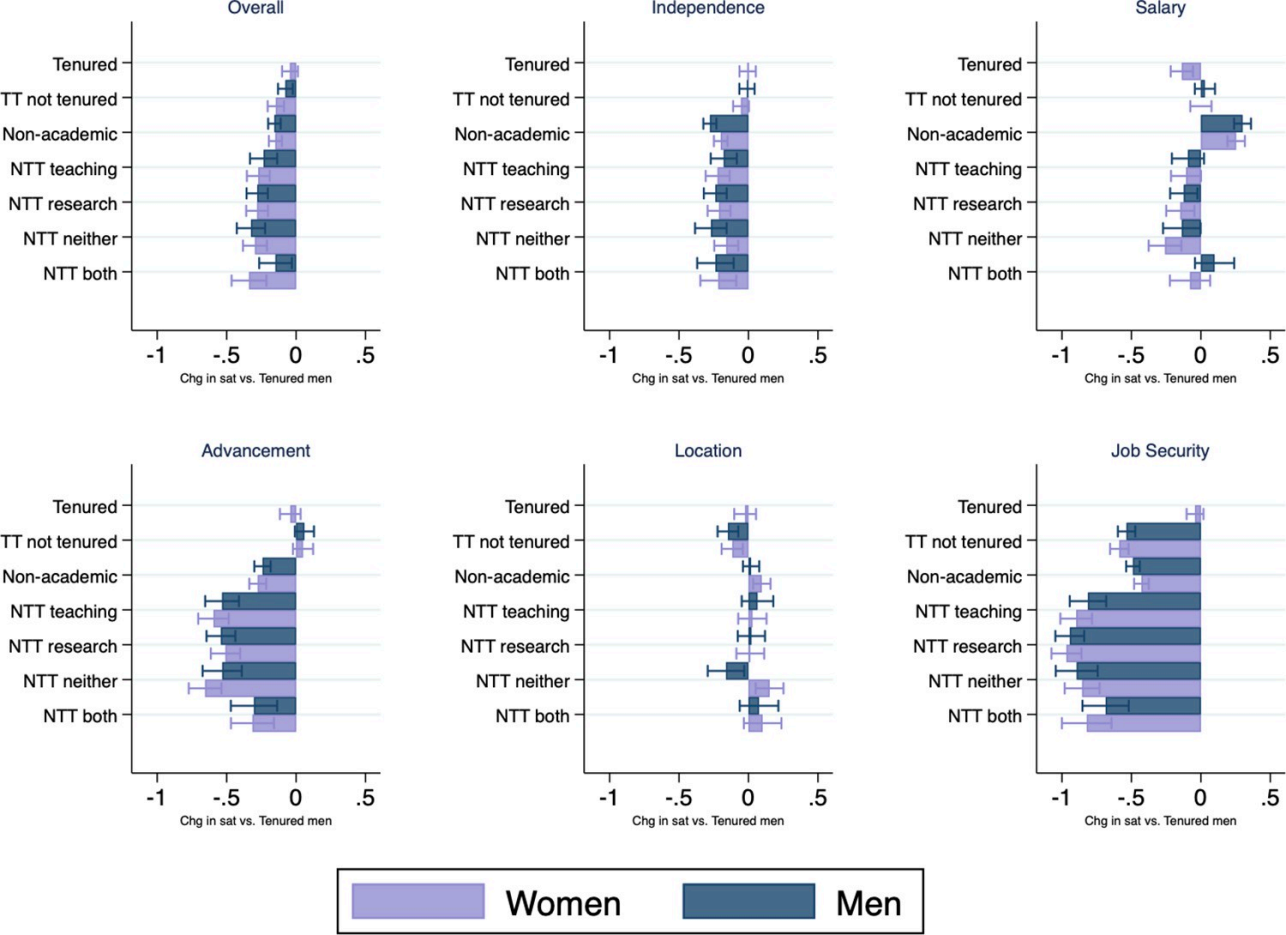
Difference in job satisfaction for various job types 7–9 years after receipt of PhD, relative to tenured men. *Notes*: Regression coefficients from a regression of job satisfaction overall (A) or with satisfaction with the specific job component listed in the subtitle (B–F) on indicators for the job types and genders as shown in the figures, as well as field of study and years since degree. Omitted category of job type and gender is tenured men, who have the highest overall job satisfaction. Analyses based on data from the National Survey of Earned Doctorates and National Survey of Doctoral Recipients.

Next, we examine the implications of holding non-tenure-track positions on women’s careers. We estimate a linear regression model to understand the trends and patterns observed in job satisfaction among men and women in different careers paths. We regress job satisfaction 7–9 years after PhD (either overall job satisfaction, or satisfaction with a particular component of the job) on indicators for men and women in various job types, controlling for field of study and years since degree. The coefficients reflect differences in job satisfaction relative to the omitted category of tenured men, who were observed to have the highest satisfaction of any gender and job type combination. Fig 2 plots the coefficients for select measures of job satisfaction for all other job type and gender combinations; coefficients for all job components are shown in S2 Table.

Both men and women who are in all types of non-tenure-track positions have significantly lower overall job satisfaction when compared to tenured men: approximately 0.2 to 0.3 points lower on a Likert scale of 1–4. Given that the average overall job satisfaction of tenured men is 3.5, this difference translates into a decrease in satisfaction of 6–9 percent for individuals in non-tenure-track positions. The magnitudes of the coefficients for men and women in all academic jobs are quite similar across all dimensions of satisfaction within job type (tenured, untenured tenure-track, and non-tenure-track), even conditional on field. These coefficients demonstrate two important patterns: (1) among those who hold academic positions, men and women do not appear to differently appreciate individual job attributes, and (2) conditional on sorting into a non-tenure-track job, men and women are equally dissatisfied. However, because women are more likely to hold these non-tenure-track positions, this results in overall lower job satisfaction among women in STEM, on average.

When looking at the various job components, those in non-tenure-track jobs have the lowest satisfaction with job security, advancement, and independence. The difference in satisfaction with job security is largest for those in non-tenure-track positions: approximately 0.8 to 0.9 points lower, or a decline of 22–25 percent relative to average satisfaction with job security among tenured men. The differences in satisfaction with advancement represent a 16–20 percent decline relative to the average satisfaction with advancement among tenured men. Those in non-tenure-track jobs who do not report either teaching or research as a primary activity are among the least satisfied across all job components.

One important exception to greater satisfaction on the tenure track is satisfaction with location, where women in non-tenure-track positions with neither teaching nor research are significantly more satisfied with their location than even tenured men (approximately 5 percent higher than the average for tenured men). This higher satisfaction with location could suggest that some women could be sorting into these positions based on geographic preferences. Looking beyond non-tenure-track positions, women and men in non-academic positions are more satisfied with their salaries (9–10 percent higher than tenured men, respectively), and women in non-academic positions are also more satisfied with their location (approximately 3 percent higher than tenured men). Strikingly, Fig 2 and S2 Table show that women who are tenured experience similar levels of satisfaction as their male tenured colleagues on all dimensions except salary, where women have lower levels of satisfaction.

**Fig 3:**
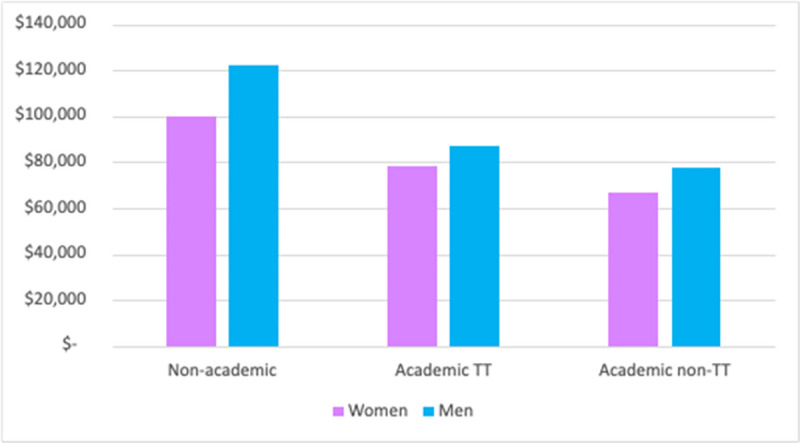
Average salaries for men and women 7–9 years after graduation, by job type. *Notes*: Analyses based on data from the National Survey of Earned Doctorates and National Survey of Doctoral Recipients.

Finally, Fig 3 compares average salaries (in $2015) for women and men by various job types, 7–9 years after receipt of the PhD. First of note is that the well-documented gender gap in salaries [19, 20] persists across all job types, and is in fact largest for non-academic careers, with an average of approximately $97,000 for women vs. $118,000. However, non-tenure-track academic salaries are lowest overall, and significantly lower for women than for men even within this group ($63,000 for women vs. $72,000 for men). In other words, the differential sorting of women into non-tenure-track positions further exacerbates the pre-existing pay gaps experienced across STEM fields overall. This gender gap in salaries persists after we adjust salaries for differences across fields (see S3 Table).

In Table 3, we test whether factors including field of study, differences in preparation during graduate school, or family characteristics can explain the observed sorting into non-tenure-track careers. We regress an indicator for employment in a non-tenure-track academic position 7–9 years after graduation on an indicator for gender, and sequentially add additional explanatory variables for each type of factor. Without controls, women are 6 percentage points more likely to be employed in these types of positions. Once we control for field of study, the gender difference in non-tenure-track academic positions falls to 4.6 percentage points, meaning the sorting of women into different fields than men (e.g., women are better represented in biology than they are in computer science) explain about a third of the overall difference. However, neither characteristics related to PhD institution and preparation nor family formation (marriage and/or having children at home) can explain the remaining gender gap in non-academic positions: it remains persistent at 4.6 percentage point difference.

**Table 3:** Regression-adjusted difference in the share of men and women in non-tenure-track academic positions.

	(1)	(2)	(3)	(4)
	All	All	All	All
Female	0.060**	0.047**	0.046**	0.046**
	(0.006)	(0.007)	(0.007)	(0.008)
Controls for field of study		X	X	X
Controls for institution quality			X	X
Controls for family structure				X
Observations	14,581	14,581	14,581	9,494
R-squared	0.006	0.014	0.014	0.026
Y-Mean	0.174	0.174	0.174	0.174

*Notes*: Dependent variable in each regression was an indicator for being in a non-tenure-track academic position. Key independent variable was an indicator for gender, reflecting the difference in the share of men and women in non-tenure-track positions. Each column in the table adds subsequent controls for field of study, preparation in graduate school, and family structure. Analyses based on data from the National Survey of Earned Doctorates and National Survey of Doctoral Recipients. Regression also controls for field, years since PhD completion and salary. Robust standard errors in parentheses.

** p<0.01

* p<0.05, + p<0.1.

We then explore if these explanations carry different weight by gender (Table 4). Seven to nine years after graduation, we observe some similar findings. Women are still more likely to work in non-tenure-track positions, even after accounting for field, and the number of years to finish a PhD remains a predictor for working in a non-tenure-track position. However, marital status is no longer a significant predictor for women. Having a degree in Biological or Life Sciences tends to be associated with lower chances of being in a non-tenure-track position at the first observation in the SDR (perhaps due to this field’s higher frequency of postdocs) but with higher chances seven to nine years after receiving the PhD. Note that this field difference is statistically significant at the *α* = .05 level only for women. Both male and female Engineering doctorates are less likely to hold non-tenure-track jobs. Having children at home marginally decreases the odds that a male doctorate is in a non-tenure-track job, but it makes no significant difference for women. These results echo a study of exits from the STEM baccalaureate workforce by Hunt, which found that family-related constraints are only a secondary factor compared to dissatisfaction with pay and promotion opportunities. [21]

**Table 4:** Regression exploring factors that may drive participation in NTT jobs, by gender.

	(1)	(2)	(3)	(4)	(5)	(6)
	Female	Female	Female	Male	Male	Male
Biological and life sciences = 1	0.056**	0.056**	0.080**	0.011	0.012	0.024
	(0.016)	(0.016)	(0.019)	(0.014)	(0.014)	(0.017)
Computer and information sciences = 1	-0.058+	-0.058+	-0.029	-0.033	-0.031	-0.059*
	(0.033)	(0.033)	(0.040)	(0.021)	(0.021)	(0.026)
Engineering = 1	-0.057**	-0.057**	-0.033	-0.069**	-0.067**	-0.059**
	(0.020)	(0.020)	(0.025)	(0.014)	(0.014)	(0.017)
Health = 1	0.015	0.015	0.016	0.043+	0.044*	0.033
	(0.020)	(0.020)	(0.026)	(0.023)	(0.023)	(0.029)
Mathematics and statistics = 1	-0.010	-0.009	0.025	-0.026	-0.027	-0.010
	(0.029)	(0.029)	(0.038)	(0.021)	(0.021)	(0.025)
Physical sciences = 1	0.004	0.004	0.035	0.000	0.001	0.040*
	(0.019)	(0.019)	(0.023)	(0.015)	(0.015)	(0.019)
Psychology = 1	0.003	0.003	0.045*	0.003	0.002	0.034
	(0.017)	(0.017)	(0.021)	(0.020)	(0.020)	(0.024)
Number of publications per faculty member at PhD Dept			-0.004			-0.003
			(0.004)			(0.003)
Number of years in PhD program			0.009**			0.008**
			(0.001)			(0.001)
Received funding through research assistantship in PhD			-0.010			-0.001
			(0.014)			(0.012)
Currently married		0.005	0.005		-0.005	0.002
		(0.013)	(0.016)		(0.012)	(0.015)
Currently has children living in home		-0.007	-0.000		-0.029**	-0.024*
		(0.011)	(0.013)		(0.009)	(0.011)
Constant	0.195**	0.195**	0.104**	0.162**	0.182**	0.098**
	(0.013)	(0.015)	(0.026)	(0.012)	(0.015)	(0.023)
Observations	6,691	6,691	4,324	7,890	7,890	5,170
R-squared	0.007	0.008	0.018	0.009	0.011	0.023
Y-Mean	0.174	0.174	0.174	0.174	0.174	0.174

*Notes*: Analyses based on data from the National Survey of Doctoral Recipients. Sample restricted to respondents 7–9 years after graduation who earned their PhD after 2000. Regression also controls for field, years since PhD completion and salary. Robust standard errors in parentheses.

** p<0.01

* p<0.05, + p<0.1.

## Discussion

Our work provides a new piece of evidence to understand one dimension of career pathways for STEM doctorate men and women that has been broadly overlooked by most of the existing literature: women are particularly disproportionately represented in non-tenure-track academic jobs. Our findings are consistent with a narrower study of ecology PhDs, which also identified an over-representation of women in non-tenure-track (excluding postdoctoral) academic jobs [22]. Interestingly, we find few field-specific differences in the likelihood of being in a non-tenure-track job: female biology doctorates are consistently more likely to be in a non-tenure-track position 7 to 9 years after graduation, and all engineering doctorates are consistently less likely to be in a non-tenure-track position (engineering doctorates are much more likely to hold nonacademic positions compared to other fields; see S1 Table for details).

The potential implications of women being disproportionately in non-tenure-track positions are mixed. First of all, non-tenure-track positions include a variety of jobs, ranging from adjunct lecturers to research scientists in academic labs, as well as management or staff positions. Some of these jobs may enable women to innovate and contribute new research to their fields, particularly those jobs in research labs. Other roles with a lot of teaching activity enable them to have direct impact on students and serve as role models. Some may come with substantial job stability; others are tenuous one-year contracts. At the same time, our tabulations show that in general, both men and women in non-tenure-track positions have significantly lower salaries, report lower overall job satisfaction, and particularly low levels of satisfaction with their opportunities for advancement and job security. Despite the fact that men and women are similarly (dis)satisfied with most aspects of their non-tenure-track jobs, there is differential sorting into these jobs, and this could be one factor driving women in STEM to be less well off in their career trajectories overall.

It is imperative to better understand the reasons behind the sorting of men and women onto different career paths even though they hold identical degrees. Based on the available measures in our dataset, men and women in non-tenure-track jobs have similar preparation during graduate school in terms of years spent in graduate school, funding and research experience in graduate school. They also face similar job options after PhD and have similar family structures. We find that observable factors that could contribute to sorting account for approximately 35 percent of the gender gap in non-tenure-track positions. As a result, the difference is likely driven by factors remaining in the residual after accounting for these factors, which could include attributes of both individuals and jobs. One clue from our analysis is the finding that higher shares of women also report holding non-tenure-track positions that are outside of their field due to a lack of other available jobs, suggesting women could be contributing less to innovation and development of future STEM scholars than they intended to due to constraints in the job market. It is also possible that academia seeks to improve the diversity of their faculty, and in order to achieve this, departments disproportionately hire women into non-tenure-track positions. Differences in workplace culture or gender discrimination are not readily discernible from the SDR data but offer additional hypotheses that we are unable to test in this study.

There are several limitations to this study. We lack sophisticated measures of researcher quality (as well as more simplistic ones like publication productivity), instead using very coarse measures of program quality. These program quality measures are also often assessed at the institution level, and neglect heterogeneity in prestige and quality within institutions. As a result, we are unable to observe specific measures of an individual graduate’s performance during graduate school or afterwards. Finally, this is not a causal study—men and women were not randomly assigned to PhD experiences (i.e., to fellowships or research assistantships, or to institutions of differing prestige), so we cannot differentiate between selection driven by individual preferences and selection driven by institutional factors. Nonetheless, the uneven distribution of women in non-tenure-track academic positions could have consequences for women’s careers in STEM, as well as for society by impacting the future STEM workforce and the extent of innovation by STEM researchers.

## Supporting information

S1 Table Job type distribution by gender and field. *Notes*: Analyses based on data from the National Survey of Earned Doctorates and National Survey of Doctoral Recipients. Sample restricted to respondents who earned their PhD after 2000. Means calculated using survey weights. The values in grey cells were suppressed due to small sample sizes. Results from first observation in SDR should not be interpreted as immediately after graduation, as individuals observed at first observation in SDR come from a variety of points in time post-graduation (e.g., some are before 3–5 years, some are after).
(DOCX)

S2 Table Associations between job satisfaction, job type, and gender, 7–9 years after PhD completion. *Notes*: Analyses based on data from the National Survey of Doctoral Recipients. Sample restricted to respondents who earned their PhD after 2000. Regression also controls for field and years since PhD completion. Robust standard errors in parentheses. Coefficients show changes in satisfaction for a given group relative to the omitted category, which is male tenured academics. ** p<0.01, * p<0.05, + p<0.1.
(DOCX)

S3 Table Job outcomes and activities by job type and gender. SDR stats measured for observations between 7–9 years post-PhD. *Notes*: Analyses based on data from the National Survey of Earned Doctorates and National Survey of Doctoral Recipients. Sample restricted to respondents who earned their PhD after 2000. Means calculated using survey weights. P-values reported from a t-test of whether the difference in means between men and women is statistically significant.
(DOCX)
