# Supplementary material for: Leak or link? the overrepresentation of women in non-tenure-track academic positions in STEM
Source: PLoS One. 2022 Jun 8;17(6):e0267561. doi: 10.1371/journal.pone.0267561 (PMC9176805; doi:10.1371/journal.pone.0267561)
Supplement: S1 Table — Notes: Analyses based on data from the National Survey of Earned Doctorates and National Survey of Doctoral Recipients. Sample restricted to respondents who earned their PhD after 2000. Means calculated using survey weights. The values in grey cells were suppressed due to small sample sizes. Results from first observation in SDR should not be interpreted as immediately after graduation, as individuals observed at first observation in SDR come from a variety of points in time post-graduation (e.g., some are before 3–5 years, some are after). (DOCX) [file pone.0267561.s001.docx]

Table S1.

Job Type Distribution by Gender and Field.

|  | Overall | | | | | | Bio/Life Science | | | | | | Computer/Info Science | | | | | |  |
| --- | --- | --- | --- | --- | --- | --- | --- | --- | --- | --- | --- | --- | --- | --- | --- | --- | --- | --- | --- |
|  | Women | | Men | | P-value | | Women | | Men | | P-value | | Women | | Men | | P-value | |  |
| **3-5 years after graduation** |  | |  | |  | |  | |  | |  | |  | |  | |  | |  |
| Academic - TT | 0.22 | | 0.21 | | 0.05 | | 0.11 | | 0.13 | | 0.060 | | 0.29 | | 0.25 | | 0.419 | |  |
| Academic, not TT (overall) | 0.22 | | 0.14 | | 0.00 | | 0.23 | | 0.18 | | <0.001 | | 0.13 | | 0.09 | | 0.110 | |  |
| Academic - not TT, teaching | 0.06 | | 0.03 | | 0.00 | | 0.04 | | 0.02 | | <0.001 | | 0.05 | | 0.02 | | 0.069 | |  |
| Academic - not TT, research | 0.07 | | 0.06 | | 0.00 | | 0.10 | | 0.10 | | 0.873 | | 0.04 | | 0.05 | | 0.898 | |  |
| Academic - not TT, both | 0.02 | | 0.02 | | 0.74 | | 0.02 | | 0.01 | | 0.440 | |  | |  | | 0.770 | |  |
| Academic - not TT, neither | 0.07 | | 0.03 | | 0.00 | | 0.07 | | 0.05 | | 0.010 | |  | |  | | 0.029 | |  |
| Postdoc | 0.09 | | 0.10 | | 0.43 | | 0.21 | | 0.23 | | 0.083 | |  | |  | | 0.289 | |  |
| Non-Academic | 0.47 | | 0.56 | | 0.00 | | 0.44 | | 0.45 | | 0.475 | | 0.55 | | 0.64 | | 0.049 | |  |
| Salary | 73,855 | | 86,734 | | 0.00 | | 67,900 | | 73,458 | | 0.000 | | 95,986 | | 116,630 | | 0.000 | |  |
| Observations | 7,281 | | 8,219 | |  | | 2,398 | | 2,107 | |  | | 198 | | 438 | |  | |  |
| **7-9 years after graduation** |  | |  | |  | |  | |  | |  | |  | |  | |  | |  |
| Academic - TT | 0.28 | | 0.28 | | 0.31 | | 0.20 | | 0.26 | | <0.001 | | 0.33 | | 0.29 | | 0.391 | |  |
| Academic, not TT (overall) | 0.20 | | 0.13 | | 0.00 | | 0.27 | | 0.18 | | <0.001 | | 0.11 | | 0.11 | | 0.961 | |  |
| Academic - not TT, teaching | 0.06 | | 0.04 | | 0.00 | | 0.05 | | 0.03 | | 0.002 | | 0.04 | | 0.04 | | 0.974 | |  |
| Academic - not TT, research | 0.07 | | 0.06 | | 0.02 | | 0.11 | | 0.09 | | 0.094 | | 0.03 | | 0.04 | | 0.840 | |  |
| Academic - not TT, both | 0.02 | | 0.01 | | 0.02 | | 0.02 | | 0.02 | | 0.112 | |  | |  | | 0.786 | |  |
| Academic - not TT, neither | 0.06 | | 0.03 | | 0.00 | | 0.08 | | 0.03 | | <0.001 | |  | |  | | 0.434 | |  |
| Postdoc | 0.02 | | 0.02 | | 0.19 | | 0.04 | | 0.06 | | 0.032 | |  | |  | | 0.562 | |  |
| Non-Academic | 0.49 | | 0.57 | | 0.00 | | 0.49 | | 0.51 | | 0.269 | | 0.54 | | 0.59 | | 0.351 | |  |
| Salary | 85,020 | | 102,410 | | 0.00 | | 81,120 | | 99,029 | | <0.001 | | 108,979 | | 127,523 | | 0.015 | |  |
| Observations | 6,691 | | 7,890 | |  | | 2,020 | | 1,921 | |  | | 181 | | 383 | |  | |  |
|  | |  | | | | | |  | | | | | |  | | | | | |
|  | | Engineering | | | | | | Health | | | | | | Math/Statistics | | | | | |
|  | | Women | | Men | | P-value | | Women | | Men | | P-value | | Women | | Men | | P-value | |
| **3-5 years after graduation** | |  | |  | |  | |  | |  | |  | |  | |  | |  | |
| Academic - TT | | 0.18 | | 0.14 | | 0.008 | | 0.31 | | 0.29 | | 0.434 | | 0.41 | | 0.39 | | 0.705 | |
| Academic, not TT (overall) | | 0.11 | | 0.08 | | 0.080 | | 0.25 | | 0.15 | | <0.001 | | 0.22 | | 0.15 | | 0.030 | |
| Academic - not TT, teaching | | 0.02 | | 0.01 | | 0.745 | | 0.08 | | 0.05 | | 0.131 | | 0.09 | | 0.06 | | 0.231 | |
| Academic - not TT, research | | 0.05 | | 0.04 | | 0.345 | | 0.08 | | 0.03 | | 0.003 | | 0.03 | | 0.03 | | 0.936 | |
| Academic - not TT, both | | 0.01 | | 0.01 | | 0.446 | | 0.03 | | 0.04 | | 0.356 | | 0.04 | | 0.05 | | 0.353 | |
| Academic - not TT, neither | | 0.03 | | 0.02 | | 0.017 | | 0.06 | | 0.02 | | 0.009 | |  | |  | | <0.001 | |
| Postdoc | | 0.06 | | 0.05 | | 0.272 | | 0.03 | | 0.05 | | 0.248 | | 0.07 | | 0.06 | | 0.653 | |
| Non-Academic | | 0.65 | | 0.73 | | <0.001 | | 0.40 | | 0.51 | | 0.000 | | 0.30 | | 0.40 | | 0.024 | |
| Salary | | 94,112 | | 101,190 | | 0.025 | | 84,278 | | 92,738 | | 0.064 | | 79,836 | | 83,856 | | 0.516 | |
| Observations | | 742 | | 2,160 | |  | | 704 | | 316 | |  | | 234 | | 406 | |  | |
| **7-9 years after graduation** | |  | |  | |  | |  | |  | |  | |  | |  | |  | |
| Academic - TT | | 0.22 | | 0.16 | | 0.002 | | 0.40 | | 0.38 | | 0.727 | | 0.49 | | 0.46 | | 0.458 | |
| Academic, not TT (overall) | | 0.12 | | 0.08 | | 0.032 | | 0.22 | | 0.20 | | 0.554 | | 0.19 | | 0.12 | | 0.03 | |
| Academic - not TT, teaching | | 0.04 | | 0.02 | | 0.008 | | 0.08 | | 0.06 | | 0.428 | | 0.11 | | 0.06 | | 0.045 | |
| Academic - not TT, research | | 0.03 | | 0.04 | | 0.393 | | 0.05 | | 0.05 | | 0.949 | | 0.03 | | 0.03 | | 0.941 | |
| Academic - not TT, both | | 0.02 | | 0.01 | | 0.475 | | 0.03 | | 0.04 | | 0.246 | |  | |  | | 0.387 | |
| Academic - not TT, neither | | 0.03 | | 0.01 | | 0.007 | | 0.06 | | 0.05 | | 0.295 | |  | |  | | 0.002 | |
| Postdoc | | 0.01 | | 0.01 | | 0.022 | |  | |  | | 0.598 | |  | |  | | 0.947 | |
| Non-Academic | | 0.65 | | 0.75 | | <0.001 | | 0.38 | | 0.40 | | 0.442 | | 0.32 | | 0.41 | | 0.020 | |
| Salary | | 103,448 | | 115,936 | | <0.001 | | 95,436 | | 102,664 | | 0.032 | | 91,423 | | 103,708 | | 0.354 | |
| Observations | | 689 | | 2,002 | |  | | 663 | | 340 | |  | | 244 | | 411 | |  | |
|  | |  | | | | | |  | | | | | |  | | | | | |
|  | |  | | | | | |  | | | | | |  | | | | | |
|  | |  | | | | | |  | | | | | |  | | | | | |
|  | |  | | | | | |  | | | | | |  | | | | | |
|  | |  | | | | | |  | | | | | |  | | | | | |
|  | | Physical Science | | | | | | Psychology | | | | | | Social Science | | | | | |
|  | | Women | | Men | | P-value | | Women | | Men | | P-value | | Women | | Men | | P-value | |
| **3-5 years after graduation** | |  | |  | |  | |  | |  | |  | |  | |  | |  | |
| Academic - TT | | 0.19 | | 0.16 | | 0.064 | | 0.19 | | 0.21 | | 0.328 | | 0.41 | | 0.47 | | 0.006 | |
| Academic, not TT (overall) | | 0.17 | | 0.13 | | <0.001 | | 0.24 | | 0.23 | | 0.490 | | 0.24 | | 0.18 | | <0.001 | |
| Academic - not TT, teaching | | 0.05 | | 0.03 | | 0.002 | | 0.07 | | 0.07 | | 0.899 | | 0.10 | | 0.09 | | 0.267 | |
| Academic - not TT, research | | 0.07 | | 0.06 | | 0.585 | | 0.05 | | 0.06 | | 0.727 | | 0.04 | | 0.03 | | 0.267 | |
| Academic - not TT, both | | 0.01 | | 0.01 | | 0.519 | | 0.02 | | 0.03 | | 0.261 | | 0.02 | | 0.01 | | 0.541 | |
| Academic - not TT, neither | | 0.04 | | 0.02 | | 0.009 | | 0.10 | | 0.07 | | 0.048 | | 0.08 | | 0.05 | | 0.017 | |
| Postdoc | | 0.11 | | 0.13 | | 0.470 | | 0.03 | | 0.03 | | 0.906 | | 0.02 | | 0.02 | | 0.850 | |
| Non-Academic | | 0.52 | | 0.59 | | 0.002 | | 0.54 | | 0.53 | | 0.884 | | 0.33 | | 0.32 | | 0.669 | |
| Salary | | 73,996 | | 84,978 | | <0.001 | | 72,266 | | 77,238 | | 0.315 | | 70,232 | | 82,331 | | <0.001 | |
| Observations | | 922 | | 1,492 | |  | | 1,241 | | 477 | |  | | 1,126 | | 924 | |  | |
| **7-9 years after graduation** | |  | |  | |  | |  | |  | |  | |  | |  | |  | |
| Academic - TT | | 0.26 | | 0.24 | | 0.487 | | 0.22 | | 0.30 | | <0.001 | | 0.46 | | 0.50 | | 0.033 | |
| Academic, not TT (overall) | | 0.17 | | 0.13 | | 0.020 | | 0.19 | | 0.15 | | 0.060 | | 0.19 | | 0.16 | | 0.130 | |
| Academic - not TT, teaching | | 0.05 | | 0.03 | | 0.009 | | 0.05 | | 0.04 | | 0.260 | | 0.07 | | 0.07 | | 0.861 | |
| Academic - not TT, research | | 0.07 | | 0.07 | | 0.757 | | 0.05 | | 0.04 | | 0.489 | | 0.04 | | 0.03 | | 0.224 | |
| Academic - not TT, both | | 0.01 | | 0.01 | | 0.246 | | 0.02 | | 0.02 | | 0.567 | |  | |  | | 0.718 | |
| Academic - not TT, neither | | 0.03 | | 0.03 | | 0.315 | | 0.07 | | 0.05 | | 0.313 | |  | |  | | 0.120 | |
| Postdoc | | 0.02 | | 0.02 | | 0.477 | | 0.01 | | 0.01 | | 0.077 | |  | |  | | 0.446 | |
| Non-Academic | | 0.55 | | 0.60 | | 0.035 | | 0.58 | | 0.53 | | 0.038 | | 0.35 | | 0.33 | | 0.265 | |
| Salary | | 85,362 | | 96,546 | | <0.001 | | 78,067 | | 96,522 | | <0.001 | | 84,376 | | 97,037 | | <0.001 | |
| Observations | | 899 | | 1,509 | |  | | 1,267 | | 491 | |  | | 1,058 | | 929 | |  | |

*Notes*: Analyses based on data from the National Survey of Earned Doctorates and National Survey of Doctoral Recipients. Sample restricted to respondents who earned their PhD after 2000. Means calculated using survey weights. The values in grey cells were suppressed due to small sample sizes. Results from first observation in SDR should not be interpreted as immediately after graduation, as individuals observed at first observation in SDR come from a variety of points in time post-graduation (e.g., some are before 3-5 years, some are after).
