# Supplementary material for: Leak or link? the overrepresentation of women in non-tenure-track academic positions in STEM
Source: PLoS One. 2022 Jun 8;17(6):e0267561. doi: 10.1371/journal.pone.0267561 (PMC9176805; doi:10.1371/journal.pone.0267561)
Supplement: S2 Table — Notes: Analyses based on data from the National Survey of Doctoral Recipients. Sample restricted to respondents who earned their PhD after 2000. Regression also controls for field and years since PhD completion. Robust standard errors in parentheses. Coefficients show changes in satisfaction for a given group relative to the omitted category, which is male tenured academics. ** p<0.01, * p<0.05, + p<0.1. (DOCX) [file pone.0267561.s002.docx]

Table S2.

Associations between job satisfaction, job type, and gender, 7-9 years after PhD completion.

|  | (1) | (2) | (3) | (4) | (5) | (6) | (7) | (8) | (9) | (10) |
| --- | --- | --- | --- | --- | --- | --- | --- | --- | --- | --- |
|  | Overall job satisfaction | Advance-ment | Benefits | Challenge | Indepen-dence | Location | Responsibility | Salary | Job security | Social impact |
|  |  |  |  |  |  |  |  |  |  |  |
| Female non-academic | -0.149** | -0.276** | -0.039 | -0.161** | -0.200** | 0.096** | -0.081** | 0.254** | -0.428** | -0.143** |
|  | (0.024) | (0.031) | (0.029) | (0.029) | (0.025) | (0.032) | (0.027) | (0.032) | (0.027) | (0.026) |
| Female, tenured | -0.044 | -0.042 | 0.015 | 0.014 | -0.006 | -0.024 | 0.034 | -0.137** | -0.042 | 0.021 |
|  | (0.029) | (0.038) | (0.036) | (0.035) | (0.030) | (0.040) | (0.034) | (0.041) | (0.031) | (0.030) |
| Female, TT not tenured | -0.146** | 0.050 | 0.003 | -0.012 | -0.054+ | -0.117** | -0.015 | 0.000 | -0.587** | -0.032 |
|  | (0.030) | (0.037) | (0.034) | (0.034) | (0.029) | (0.039) | (0.033) | (0.039) | (0.034) | (0.030) |
| Female NTT, teaching | -0.272** | -0.595** | -0.109* | -0.307** | -0.222** | 0.028 | -0.201** | -0.107+ | -0.898** | -0.097* |
|  | (0.042) | (0.056) | (0.052) | (0.051) | (0.044) | (0.052) | (0.047) | (0.055) | (0.058) | (0.042) |
| Female NTT, research | -0.281** | -0.508** | 0.080+ | -0.028 | -0.212** | 0.013 | -0.097* | -0.148** | -0.968** | -0.148** |
|  | (0.040) | (0.054) | (0.047) | (0.043) | (0.042) | (0.051) | (0.042) | (0.052) | (0.055) | (0.041) |
| Female NTT, both teaching and research | -0.339** | -0.314** | -0.112 | 0.001 | -0.217** | 0.102 | -0.057 | -0.078 | -0.822** | -0.000 |
|  | (0.064) | (0.079) | (0.074) | (0.061) | (0.066) | (0.069) | (0.064) | (0.074) | (0.091) | (0.057) |
| Female NTT, neither | -0.296** | -0.655** | -0.238** | -0.252** | -0.160** | 0.152** | -0.172** | -0.258** | -0.855** | -0.148** |
|  | (0.044) | (0.060) | (0.063) | (0.054) | (0.044) | (0.051) | (0.051) | (0.060) | (0.064) | (0.046) |
| Female, postdoc | -0.412** | -0.620** | -0.239* | -0.007 | -0.305** | 0.076 | -0.155* | -0.446** | -1.236** | -0.230** |
|  | (0.077) | (0.106) | (0.093) | (0.069) | (0.081) | (0.088) | (0.077) | (0.099) | (0.100) | (0.068) |
| Male non-academic | -0.156** | -0.242** | 0.042 | -0.196** | -0.278** | 0.019 | -0.157** | 0.300** | -0.489** | -0.219** |
|  | (0.023) | (0.030) | (0.027) | (0.028) | (0.024) | (0.030) | (0.027) | (0.031) | (0.025) | (0.025) |
| Male, TT not tenured | -0.077** | 0.060+ | -0.017 | 0.030 | -0.011 | -0.148** | 0.005 | 0.029 | -0.535** | -0.025 |
|  | (0.027) | (0.035) | (0.033) | (0.033) | (0.028) | (0.038) | (0.031) | (0.037) | (0.032) | (0.028) |
| Male NTT, teaching | -0.234** | -0.533** | -0.044 | -0.213** | -0.178** | 0.065 | -0.151** | -0.093 | -0.813** | -0.009 |
|  | (0.050) | (0.062) | (0.057) | (0.053) | (0.048) | (0.058) | (0.052) | (0.059) | (0.067) | (0.044) |
| Male NTT, research | -0.280** | -0.541** | 0.103* | 0.002 | -0.239** | 0.021 | -0.137** | -0.123* | -0.944** | -0.176** |
|  | (0.039) | (0.053) | (0.044) | (0.043) | (0.042) | (0.050) | (0.042) | (0.050) | (0.053) | (0.041) |
| Male NTT, both teaching and research | -0.148* | -0.303** | 0.024 | -0.077 | -0.238** | 0.076 | -0.106 | 0.099 | -0.686** | -0.093 |
|  | (0.060) | (0.085) | (0.070) | (0.073) | (0.067) | (0.071) | (0.068) | (0.072) | (0.085) | (0.059) |
| Male NTT, neither | -0.325** | -0.532** | -0.264** | -0.301** | -0.271** | -0.162* | -0.211** | -0.137* | -0.894** | -0.164** |
|  | (0.052) | (0.072) | (0.073) | (0.064) | (0.058) | (0.067) | (0.059) | (0.069) | (0.077) | (0.050) |
| Male, postdoc | -0.542** | -0.650** | -0.327** | -0.111 | -0.415** | -0.077 | -0.252** | -0.493** | -1.202** | -0.327** |
|  | (0.068) | (0.086) | (0.080) | (0.072) | (0.076) | (0.074) | (0.071) | (0.086) | (0.090) | (0.066) |
|  |  |  |  |  |  |  |  |  |  |  |
| Constant | 3.530** | 3.046** | 3.254** | 3.347** | 3.647** | 3.227** | 3.316** | 2.794** | 3.663** | 3.306** |
|  | (0.069) | (0.090) | (0.084) | (0.082) | (0.073) | (0.086) | (0.077) | (0.086) | (0.087) | (0.072) |
| Observations | 14359 | 12386 | 12386 | 12386 | 12386 | 12386 | 12386 | 12386 | 12386 | 12386 |
| R-squared | 0.015 | 0.049 | 0.007 | 0.017 | 0.026 | 0.009 | 0.014 | 0.053 | 0.090 | 0.029 |

*Notes*: Analyses based on data from the National Survey of Doctoral Recipients. Sample restricted to respondents who earned their PhD after 2000. Regression also controls for field and years since PhD completion. Robust standard errors in parentheses. Coefficients show changes in satisfaction for a given group relative to the omitted category, which is male tenured academics. ** p<0.01, * p<0.05, + p<0.1
