# Supplementary material for: Leak or link? the overrepresentation of women in non-tenure-track academic positions in STEM
Source: PLoS One. 2022 Jun 8;17(6):e0267561. doi: 10.1371/journal.pone.0267561 (PMC9176805; doi:10.1371/journal.pone.0267561)
Supplement: S3 Table — SDR stats measured for observations between 7–9 years post-PhD. Notes: Analyses based on data from the National Survey of Earned Doctorates and National Survey of Doctoral Recipients. Sample restricted to respondents who earned their PhD after 2000. Means calculated using survey weights. P-values reported from a t-test of whether the difference in means between men and women is statistically significant. (DOCX) [file pone.0267561.s003.docx]

**Table S3.**

Job outcomes and activities by job type and gender. SDR stats measured for observations between 7-9 years post-PhD

|  | All | Women | Men | P-value | All | Women | Men | P-value |  |
| --- | --- | --- | --- | --- | --- | --- | --- | --- | --- |
|  | Nonacademic | | | | Academic TT - tenured | | | | |
| Work funded by grants | 0.20 | 0.20 | 0.20 | 0.926 | 0.35 | 0.30 | 0.39 | <0.001 |  |
| Primary work activity is research | 0.75 | 0.70 | 0.78 | <0.001 | 0.89 | 0.87 | 0.90 | 0.014 |  |
| Number of ppl direct supervise | 4.70 | 5.03 | 4.53 | 0.024 | 6.42 | 6.54 | 6.36 | 0.744 |  |
| # of professional meetings attended | 0.57 | 0.56 | 0.58 | 0.143 | 0.86 | 0.84 | 0.87 | 0.091 |  |
| # of professional society membership | 1.31 | 1.36 | 1.28 | 0.043 | 2.62 | 2.77 | 2.51 | 0.002 |  |
| Working outside field because other job not available | 0.40 | 0.42 | 0.39 | 0.549 | 0.22 | 0.29 | 0.20 | 0.754 |  |
| Salary, adjusted for differences across fields | 108,031 | 99,255 | 113,051 | <0.001 | 79,900 | 79,394 | 80,247 | 0.626 |  |
|  | Academic TT - not tenured | | | |  |  |  |  | |
| Work funded by grants | 0.44 | 0.40 | 0.46 | 0.003 |  |  |  |  |  |
| Primary work activity is research | 0.93 | 0.91 | 0.95 | <0.001 |  |  |  |  |  |
| Number of ppl direct supervise | 5.12 | 5.15 | 5.11 | 0.889 |  |  |  |  |  |
| # of professional meetings attended | 0.90 | 0.89 | 0.91 | 0.216 |  |  |  |  |  |
| # of professional society membership | 2.60 | 2.64 | 2.58 | 0.427 |  |  |  |  |  |
| Working outside field because other job not available | 0.14 | 0.17 | 0.12 | 0.825 |  |  |  |  |  |
| Salary, adjusted for differences across fields | 81,904 | 76,175 | 85,863 | <0.001 |  |  |  |  |  |
|  | Academic not tenure track, teaching position | | | | Academic not tenure track, research position | | | | |
| Work funded by grants | 0.11 | 0.11 | 0.11 | 0.875 | 0.72 | 0.69 | 0.74 | 0.127 |  |
| Primary work activity is research | 0.52 | 0.49 | 0.56 | 0.077 | 0.97 | 0.96 | 0.97 | 0.670 |  |
| Number of ppl direct supervise | 6.54 | 6.73 | 6.37 | 0.745 | 4.58 | 5.83 | 3.53 | 0.186 |  |
| # of professional meetings attended | 0.62 | 0.61 | 0.63 | 0.682 | 0.75 | 0.77 | 0.72 | 0.110 |  |
| # of professional society membership | 1.93 | 1.87 | 1.99 | 0.390 | 1.79 | 1.85 | 1.75 | 0.294 |  |
| Working outside field because other job not available | 0.27 | 0.13 | 0.37 | 0.245 | 0.39 | 0.41 | 0.38 | 0.885 |  |
| Salary, adjusted for differences across fields | 68,722 | 65,881 | 71,770 | 0.037 | 68,722 | 73,041 | 74,289 | 0.490 |  |

|  | |  | | | | | | | |  | |  | |  | |  | | |
| --- | --- | --- | --- | --- | --- | --- | --- | --- | --- | --- | --- | --- | --- | --- | --- | --- | --- | --- |
|  | |  | |  | |  | |  | |  | |  | |  | |  | | |
|  | Academic not tenure track, teaching & research | | | | | | | | Academic not tenure track, neither | | | | | | | | |  |
| Work funded by grants | 0.38 | | 0.37 | | 0.40 | | 0.682 | | 0.28 | | 0.24 | | 0.34 | | 0.006 | |  |  |
| Primary work activity is research | 0.92 | | 0.88 | | 0.96 | | 0.007 | | 0.51 | | 0.48 | | 0.57 | | 0.026 | |  |  |
| Number of ppl direct supervise | 5.67 | | 6.09 | | 5.37 | | 0.419 | | 6.02 | | 5.61 | | 6.53 | | 0.380 | |  |  |
| # of professional meetings attended | 0.88 | | 0.88 | | 0.87 | | 0.758 | | 0.56 | | 0.57 | | 0.53 | | 0.372 | |  |  |
| # of professional society membership | 2.55 | | 2.56 | | 2.54 | | 0.884 | | 1.58 | | 1.61 | | 1.53 | | 0.563 | |  |  |
| Working outside field because other job not available | 0.82 | | 0.92 | | 0.63 | | 0.479 | | 0.31 | | 0.39 | | 0.20 | | 0.082 | |  |  |
| Salary, adjusted for differences across fields | 85,444 | | 80,492 | | 89,933 | | 0.130 | | 65,918 | | 62,533 | | 71,010 | | 0.018 | |  |  |

*Notes*: Analyses based on data from the National Survey of Earned Doctorates and National Survey of Doctoral Recipients. Sample restricted to respondents who earned their PhD after 2000. Means calculated using survey weights. P-values reported from a t-test of whether the difference in means between men and women is statistically significant.
